# Geriatrics in Practice and Research—Worldwide

**DOI:** 10.3390/geriatrics1010001

**Published:** 2015-12-22

**Authors:** Ralf Lobmann

**Affiliations:** Department of Endocrinology, Diabetology and Geriatrics, Clinical Centre Stuttgart, Bad Cannstatt, Prießnitzweg 24, D-70374 Stuttgart, Germany; R.Lobmann@klinikum-stuttgart.de; Tel.: +49-711-278-22601; Fax: +49-711-278-22173

This edition marks the launch of the new “Geriatrics” open access journal. Clinical geriatrics and the associated scientific activities required in the field constitute one of the major scientific challenges in medicine in the future. The particular significance of this issue is not only from the fact that demographics are changing in industrialized nations, e.g., in Germany, where 37% of the population will be over 60 and 11% over 80 in 2040, and from the current projection suggesting that children born in the year 2015 have a 50% chance of reaching the age of 100 [1]. 

It is common knowledge that older and elderly people in particular place a particular burden on the resources of the health care system. Additionally, in emerging and developing nations, medical care for older people is becoming an increasingly difficult issue in view of the unique age-related needs of the elderly.

It is thus a fact that health care systems must tackle the special challenges of an aging society and treating elderly patients on a global level for medical and, in particular, economic reasons.

Furthermore, it is not only geriatric specialists that must and should address the problems of treating the elderly, but geriatric medicine overflows into most medical disciplines (e.g., orthopedics, urology, all areas of internal medicine and neurology and geriatric psychiatry).

With our new journal we will act as an interface for geriatric specialists and colleagues from other disciplines who are interested in geriatrics, as well as an interface between theory and clinical practice, which is depicted by the structure of the journal, with research articles that refer to clinical and practical reviews on the same topics, as well as in the range of Special Issues that we have planned, which are already being prepared on the topics of diabetes in older people, dementia, and delirium.

Finally, we have chosen the format of an open access journal to provide all interested parties a platform for their basic research and health care studies, as well as to encourage a wealth of feedback from our readers, who may already find themselves in such a demographic environment or who are taking early steps to prepare for demographic trends, particularly in emerging markets, as mentioned above, thereby providing them with access to scientifically well-founded and practice-oriented geriatric topics.
